# Tsetse flies (*Glossina morsitans morsitans*) choose birthing sites guided by substrate cues with no evidence for a role of pheromones

**DOI:** 10.1098/rspb.2023.0030

**Published:** 2023-04-26

**Authors:** Andrea K. Adden, Lee R. Haines, Álvaro Acosta-Serrano, Lucia L. Prieto-Godino

**Affiliations:** ^1^ Neural Circuits and Evolution Laboratory, Francis Crick Institute, London NW1 1AT, UK; ^2^ Department of Vector Biology, Liverpool School of Tropical Medicine, Liverpool L3 5QA, UK

**Keywords:** tsetse, maternal care, behaviour, *Glossina morsitans*, pheromone, ecology

## Abstract

Tsetse flies significantly impact public health and economic development in sub-Saharan African countries by transmitting the fatal disease African trypanosomiasis. Unusually, instead of laying eggs, tsetse birth a single larva that immediately burrows into the soil to pupate. Where the female chooses to larviposit is, therefore, crucial for offspring survival. Previous laboratory studies suggested that a putative larval pheromone, *n*-pentadecane, attracts gravid female *Glossina morsitans morsitans* to appropriate larviposition sites. However, this attraction could not be reproduced in field experiments. Here, we resolve this disparity by designing naturalistic laboratory experiments that closely mimic the physical characteristics found in the wild. We show that gravid *G. m. morsitans* were neither attracted to the putative pheromone nor, interestingly, to pupae placed in the soil. By contrast, females appear to choose larviposition sites based on environmental substrate cues. We conclude that, among the many cues that likely contribute to larviposition choice in nature, substrate features are a main determinant, while we failed to find evidence for a role of pheromones.

## 1. Introduction

Across animal species, a wide variety of reproductive strategies have evolved—from a reliance on large numbers of offspring with limited or non-existent maternal care (e.g. mosquitoes, [1]), to enormous maternal investment in animals, including our own species, with long pregnancies and small numbers of offspring. This latter strategy has evolved multiple times across phyla [2]. Tsetse flies (*Glossina* sp.), vectors of the African trypanosome parasites, which cause sleeping sickness and animal trypanosomiasis, provide a fascinating example. Instead of laying eggs—like most insects—each tsetse female matures one larva at a time in her uterus. The larva is sustained by feeding on ‘tsetse milk’, which is secreted by modified accessory glands [3]. After approximately 10 days *in utero*, when the larva reaches the end of its third instar stage, the female gives birth in a process known as larviposition. The larva then rapidly burrows into the ground on which it was deposited to commence pupation. The female's choice of larviposition site is thus her final maternal care behaviour, and her choice can markedly affect the offspring's chance of survival.

One indicator for favourable soil conditions may be the presence of other tsetse larvae and/or pupae, and previous work has suggested that this may be signalled by larval pheromones [4,5]. Chemical analysis of the larval exudates of two subspecies of *Glossina morsitans* identified the alkanes *n*-pentadecane (for *G. morsitans morsitans*) and *n*-dodecane (for *G. morsitans centralis*) as the major active component for each subspecies, as both attracted conspecific gravid females in a two-choice preference assay [5]. Similarly, in *Glossina brevipalpis* and *Glossina palpalis*, significant attraction was reported when sand was conditioned with conspecific as well as heterospecific larvae [6,7]. Although no larviposition pheromone was identified in these studies, the authors suggested that pheromones may be similar across these two species.

Given that tsetse have a significant impact on public health and economic development in sub-Saharan African countries [8,9], a larviposition pheromone that reliably attracts gravid females could open promising new avenues in vector control (as suggested in [10]). However, when a recent study tested the effectiveness of *n*-pentadecane as an attractant of gravid female *G. m. morsitans* in the field, it found no effects at any concentration ([11]; see also [12]). These contradictory results raise the question of whether findings from behavioural assays, performed under artificial laboratory conditions, are applicable to the complex natural ecology of tsetse in the natural environment. We aimed at closing this gap, and revisiting the evidence for the existence of a larviposition pheromone in *G. m. morsitans*, by conducting carefully controlled laboratory experiments that nevertheless presented the flies with conditions that were as naturalistic as possible—that is to say they matched as closely as possible the features of larviposition sites that a fly might encounter in the field.

Our results demonstrate that *G. m. morsitans* females display a clear preference for certain environmental cues, such as substrate consistency and shade, which is in agreement with studies that surveyed pupation sites in the field [13]. However, we found that under naturalistic settings, neither the putative *G. m. morsitans* pheromone *n*-pentadecane nor the aggregation of pupae in the substrate had any effect on the larviposition choices of these flies. We conclude that it is unlikely that larviposition in *G. m. morsitans* is guided by larval pheromones in natural ecological settings, and that other environmental cues are the major drivers of female larviposition choices.

## 2. Results

### (a) Minimizing stress in larviposition choice assays

Previous studies employed standardized laboratory methods that have the potential to induce stress in tsetse flies, yet they did not assess whether flies were stressed and if this could impact the results. In addition to the handling of the gravid females, the two most important stressors introduced in these assays were the colour of the behavioural arenas and the larviposition substrate. Two out of three studies [5,6] used white enclosures, and all studies used plain white sand as a larviposition substrate [5–7]. Bright spaces are, however, usually associated with the search for host animals, whereas females in late stages of pregnancy rarely feed, and are more likely to rest in shaded areas [14]. Furthermore, fieldwork has shown that tsetse tend to larviposit under fallen logs, in tree holes and in underground burrows, and prefer leaf litter to exposed sand [12,13,15].

To address these discrepancies, our behavioural paradigm was designed to be as naturalistic and stress-free for the flies as possible, while also being a carefully controlled insectary experiment. Non-forced two-choice experiments, in which females could choose between two options but were also free to deposit on the floor of the cage instead, were conducted in a large flight cage with brown-stained netting, and we used natural-colour sand (electronic supplementary material, figure S1*a*,*b*). We opted for a non-forced paradigm because this enables us to interpret larviposition within the stimulus/control trays as a true choice.

**Figure 1.**
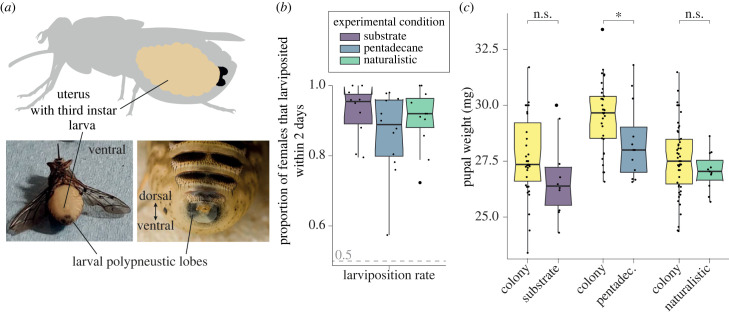
Female and larval health. (*a*) Females were selected for experiments if the polypneustic lobes of the larva were clearly visible through the female's cuticle. (*b*) Proportion of females that larviposited within 2 days (larviposition rate). Median larviposition rates for the three experimental conditions were 95.46% (substrate), 88.88% (pentadecane) and 92.00% (naturalistic). The larviposition rate was highly consistent across experimental conditions (not significant (n.s.), Kolmogorov–Smirnov test). (*c*) Pupal weight measured for all experimental conditions and compared with pupal weights measured in the colony at the time of the experiment. Median pupal weights were 26.39 mg (substrate), 28.0 mg (pentadecane) and 27.01 mg (naturalistic). Pupal weights obtained from pentadecane experiments are significantly smaller than colony weights (*p = 0.0233, Kolmogorov–Smirnov test). However, all experimental weight distributions fall within the expected weight distribution of the colony.

We evaluated female stress by (i) counting the number of abortions induced by handling, (ii) calculating the proportion of females that larviposited within 48 h (larviposition rate), and (iii) measuring the weight of the pupae as an indicator of sustained female investment (figure 1). We tested a total of 1639 female *G. m. morsitans*, which were selected for experiments if the polypneustic lobes of the larva were visible through the female's cuticle (figure 1*a*). The females produced 1423 larvae within 48 h, at an overall larviposition rate of 86.8% per 48 h, which was highly consistent across experimental conditions (figure 1*b*). The overall abortion rate was a negligible 0.43%, and the mortality rate among females was less than 2%.

As a measure of larval health, we measured the weight of all deposited pupae. For all experiments, pupal weight distributions fell within the measured distribution of the colony at the time of the experiment (figure 1*c*), although experimental pupae were slightly smaller than control pupae. However, colony records show that first-time mothers produce overall smaller larvae than older mothers, and females chosen for our experiments often included first-time mothers [16]. It is, therefore, not unexpected that median experimental weights were lower. We conclude that stress was not a significant factor in our experiments.

### (b) Tsetse prefer to larviposit on leaf litter over sand

**Figure 2.**
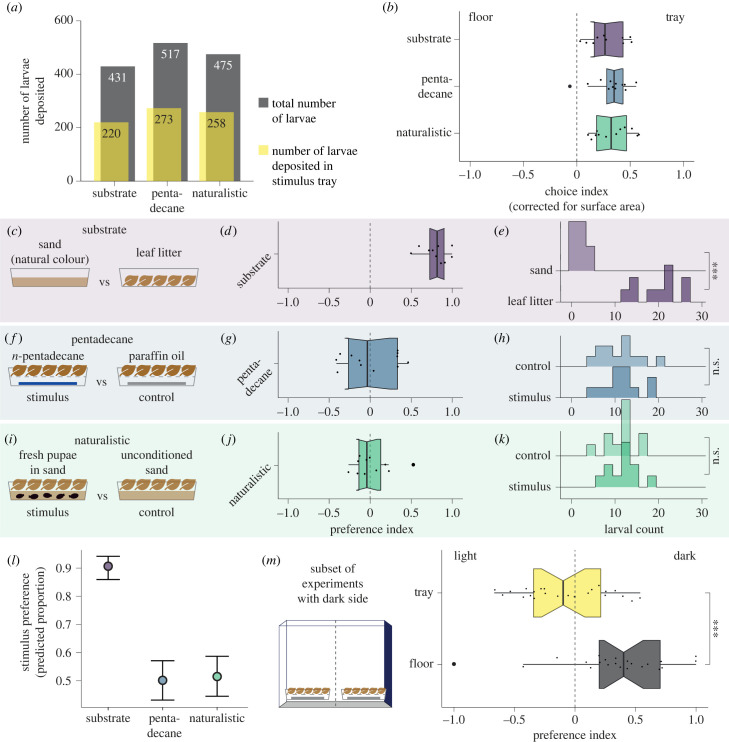
Female larviposition preferences. (*a*) Absolute number of larvae deposited during experiments. Grey: total number of larvae deposited across cages per experiment. Yellow: number of larvae deposited in stimulus trays, rather than on the floor. Larvae deposited on the floor were not included in the analysis. (*b*) Choice index for larvipositing on trays versus on the floor. The choice index was corrected for surface area. Across experiments females prefer to larviposit on trays. (*c*) In the ‘substrate’ condition, females were offered the choice between natural-colour sand and leaf litter as larviposition substrate. (*d*) The preference index (PI) for the ‘substrate’ condition shows a strong preference for leaf litter (median PI = 0.817). (*e*) A ridge histogram of larval count for each stimulus in the ‘substrate’ condition shows a significantly higher larval count in leaf litter (*p* < 0.001, Wilcoxon rank-sum test). (*f*) In the ‘pentadecane’ condition, females chose between the proposed larval pheromone *n*-pentadecane and the solvent paraffin oil as a control stimulus. (*g*) PI for the ‘pentadecane’ condition shows no preference for *n*-pentadecane. (*h*) The distributions of larval count in the stimulus and control trays strongly overlap. (*i*) In the ‘naturalistic’ condition, females chose between sand conditioned with 20 1-day-old pupae, and unconditioned sand. (*j*) PI for the ‘naturalistic’ condition shows no preference for conditioned sand. (*k*) The distributions of larval count in conditioned versus unconditioned sand strongly overlap. (*l*) Conditional effects predicted by a Bayesian model of female choice. The model supports a preference only in the ‘substrate’ condition (estimated median = 0.906, s.e. = 0.02) but not in the ‘pentadecane’ or ‘naturalistic’ conditions (estimated medians 0.502 and 0.5125, respectively). (*m*) In a subset of experiments, the cage had one dark wall. The PI for cage side shows that females depositing in trays do not have a preference for the darker side of the cage (no difference from normal distribution, mean = 0, s.d. = 1, Kolmogorov–Smirnov test), but females depositing on the floor prefer the darker half of the floor (median PI = 0.402; difference from normal distribution, mean = 0, s.d. = 1: *p* ≪ 0.001, Kolmogorov–Smirnov test).

Field experiments have reported that tsetse prefer to larviposit on leaf litter over sand [15], yet all prior laboratory larviposition assays used sand as a substrate [5–7]. To confirm which substrate is preferred by tsetse, we tested females in a non-forced two-choice larviposition assay, in which flies could choose to larviposit in trays filled with either sterilized leaf litter or sand, or on the floor (figure 2*c*). We found that when corrected for surface area, flies preferred to larviposit on the trays compared with the floor, and this held true across all experiments (figure 2*b*). Because we were interested in the choice between the two substrates, we only used larvae deposited on either tray to calculate a preference index ($\mathrm{PI}=(n_{\mathrm{stimulus}}-\;n_{\mathrm{control}})/(n_{\mathrm{stimulus}}+n_{\mathrm{control}})$, ranging from −1 (absolute preference for control) to 1 (absolute preference for stimulus)). We found a clear preference for leaf litter, with a median PI of 0.82 (figure 2*d*) and significantly higher larval counts in the leaf litter tray as compared with the sand tray (*p* < 0.001, Wilcoxon rank-sum test; figure 2*e*). Across trials, 90.46% of larvae were deposited on leaf litter. We conclude that gravid *G. m. morsitans* females prefer to larviposit on leaf litter, as suggested by previous reports from the field. This highlights that previous larviposition experiments, carried out in the laboratory, were performed with suboptimal substrates. Given these results, we chose to use leaf litter rather than sand as larviposition substrate in the following experiments.

### (c) *n*-pentadecane does not act as a larviposition pheromone for *G. m. morsitans* under naturalistic laboratory settings

We next tested whether the described larval pheromone *n*-pentadecane attracts *G. m. morsitans* females for larviposition. We presented a dilution of *n*-pentadecane on filter paper that was the equivalent of volatile released by 80 pupating larvae over 2 h—a dose that was reported to attract approximately 75% of females [5]. The *n*-pentadecane was presented on filter paper under a suspended layer of leaf litter, such that the females could not touch the *n*-pentadecane itself (figure 2*f*). As a control stimulus, the solvent paraffin oil was also presented on filter paper under leaf litter. To ensure that the leaf litter did not contain traces of *n*-pentadecane that could interfere with our experiment, all leaf litter was sterilized at 121°C to eliminate volatile compounds, and subsequent gas chromatography–mass spectrometry (GC–MS) analysis confirmed the absence of *n*-pentadecane (electronic supplementary material, figure S2). Under these conditions, we found no preference (median PI = −0.034, figure 2*g*), with 50.18% of larvae deposited in the stimulus tray. Furthermore, the distributions of larval counts in each stimulus tray strongly overlap (figure 2*h*). This suggests that *n*-pentadecane alone is not sufficient to attract *G. m. morsitans* to a larviposition site.

### (d) No evidence for the presence of a larviposition pheromone in tsetse

We reasoned that although *n*-pentadecane did not act as a larviposition pheromone, other compounds emitted by pupating larvae might contribute to an attractive effect. To test for a putative larviposition pheromone, we devised a naturalistic experiment in which 20 1-day-old pupae were presented in sand (where they had pupated) under a layer of leaf litter, again so the flies were unable to make contact with the sand (figure 2*i*). The control stimulus was sand that did not contain any pupae, presented under leaf litter. Under these conditions, females again showed no preference (PI = −0.04, figure 2*j*), with 50.78% of pupae deposited in the stimulus tray. As in the ‘pentadecane’ condition, the larval count distributions strongly overlap (figure 2*k*). We therefore conclude that under naturalistic conditions, larval and pupal volatiles are not sufficient to attract *G. m. morsitans* to a larviposition site. These results are supported by a generalized linear mixed-effects model of experimental conditions using Bayesian inference, which supports a preference only in the ‘substrate’ condition but not in the ‘pentadecane’ or ‘naturalistic’ conditions (figure 2*l*).

### (e) Shade is preferred but is secondary to substrate quality

Another cue that has been proposed to be important for larviposition preference in the field is the presence of shade [15]. Although we did not test this directly, one set of experiments was carried out in cages where one cage wall was darker than the others (see Methods). When we analysed these results, we found that the number of larvae deposited on the trays filled with leaf litter did not depend on whether the trays were oriented to the dark or light side of the cage (figure 2*m*). However, for larvae deposited on the floor, there was a clear preference for the dark side of the cage (figure 2*m*). These results indicate that, while substrate quality is a key larviposition cue, a darker environment is preferred in the absence of other cues, something that has also been suggested previously [12].

## 3. Discussion

An outstanding question in tsetse ecology was whether these viviparous flies use pheromones to guide their larviposition choices. Three laboratory studies argued this to be the case, and one study identified the putative pheromone compound for *G. m. morsitans*, *n*-pentadecane [5–7]. However, a recent study in the field was unable to reproduce these results [11]. Here we bridge the gap between larviposition experiments in the laboratory and in the field. We have shown that it is possible to undertake naturalistic larviposition experiments under tightly controlled laboratory conditions, allowing us to reproduce results from the field. Our results suggest that the prior discrepancies between laboratory and field studies may be due to artificial conditions imposed on gravid females in laboratory settings. Importantly, our work paves the way for future behavioural experiments in the laboratory under more naturalistic conditions.

We show that under naturalistic conditions in the laboratory, pheromones do not seem to play a significant role in female larviposition choice in the savannah tsetse *G. m. morsitans*, and that environmental cues appear to be the main drivers of this choice. Among environmental cues, larviposition substrate seems to be the most important one, with females clearly preferring to larviposit on leaf litter compared with sand. Furthermore, we show that shaded environments are also preferred, which is consistent with prior field data [11,15]. Leaf litter has a well-described function of shielding the soil surface from rapid changes in temperature and humidity, thus helping to create a stable environment for soil-living organisms [17]. Rates of pupal development in tsetse are strongly temperature-dependent [18,19] and gravid females are known to seek out shaded and sheltered areas such as warthog burrows to deposit their larvae [15], where temperature fluctuations are minimized. The presence of leaf litter alone is, therefore, likely to signal appropriate larviposition sites, both in terms of maternal care by selecting optimal conditions for larval development, and in providing shade and shelter for the female and her larva during parturition.

While we confirm that gravid *G. m. morsitans* strongly prefer leaf litter, previous laboratory studies provided sand as a larviposition substrate [5–7]. The sand used in these studies was white, in contrast to the natural-coloured sand we used in the present study. It is well described that tsetse are not attracted to white surfaces [20,21]. Therefore, the brightness of the white sand may have added to the artificial nature of the experiments. In the field, flies tend to rest in the shade, e.g. on the underside of tree branches, throughout the hottest part of the day [14]. We conclude that factors such as shade can strongly influence the flies in the absence of other cues, as previously reported [12]. It is clear that in future studies, the light/shade distribution in the cages needs to be taken into account and, if possible, controlled for.

Our finding that neither *n*-pentadecane nor other potential pheromones appear to guide larviposition decisions in *G. m. morsitans* under naturalistic conditions raises further questions, especially considering that tsetse pupae are often found aggregated in the field [6,12,22]. Although a pheromone could mark a site at which other larvae have successfully pupated, thus overcoming environmental obstacles such as desiccation and overheating [19], tsetse pupae are at constant risk of predation by ants and beetles [23–25], and a volatile pheromone strong enough to attract females may also attract predators. As *n*-pentadecane is only released during early pupation [4], but pupal development takes approximately three to four weeks in *G. m. morsitans*, *n*-pentadecane would carry no information about the subsequent survival of the pupae. Interestingly, *n*-pentadecane has antimicrobial properties [26], and it is conceivable that the exudates from aggregated pupae create a microbicidal microenvironment in which pupae are more likely to develop optimally and survive. While there could, therefore, be strength in numbers, pupal aggregation exceeding a critical density threshold can be detrimental to survival owing to higher predation [27].

In the field, leaf litter releases a substantial number of volatiles, potentially including *n*-pentadecane—a known volatile of many plant species. We therefore favour the proposal of Hargrove *et al*. [11] that in natural settings, pentadecane of botanical origin will likely mask larval volatiles. As a consequence, larval *n*-pentadecane emissions may be of practical use to females only under very specific circumstances, e.g. when larviposition sites are limited and bare soil or sand are the only options. This is in agreement with multiple field studies that have established that in the wild tsetse select larviposition sites based on season, temperature and soil conditions [12,15,23,24].

Thus, the advantages of pupal aggregation remain unclear, as do the mechanisms that attract female tsetse to the same larviposition sites.

To clarify the mechanisms that underlie female attraction to leaf litter, future studies should focus on analysing the chemical profile of leaf litter found in the natural habitat of *G. m. morsitans*, to identify additional potential attractants. It will also be interesting to disentangle the relative contributions of different senses, e.g. olfactory versus visual, to larviposition site choice in the natural environment. Furthermore, while we tested the equivalent concentrations of *n*-pentadecane released by 20 and 80 pupating larvae, respectively, we cannot rule out that higher (or lower) concentrations may have had an attractive effect on females. Testing additional doses of the pheromone was outside the scope of this study, but Hargrove *et al*. [11] tested a wider range of concentrations in the field without finding an attractive effect. We therefore do not expect that our results and conclusions would differ significantly if we had tested additional concentrations. Of course, the possibility remains that other tsetse species rely more heavily on larval pheromones when choosing larviposition sites—the hierarchy of cues driving this behaviour may differ depending on species and habitat. Future studies under naturalistic conditions in the field will be necessary to completely understand this cue hierarchy across species.

To conclude, this study demonstrates the importance of taking an animal's natural environment into account when designing laboratory experiments with colony-reared insects. In the case of tsetse, new attractants found in the laboratory understandably create much interest as potential new ways of vector control. While carefully controlled laboratory studies can generate exciting new insights into tsetse biology, these insights may not be relevant when taking the flies' ecology and natural environment into account, and attractants identified in the laboratory may indeed be overridden by other environmental cues. We therefore recommend that future insectary-based studies match the natural environment as far as possible.

## 4. Material and methods

### (a) Animals

*Glossina morsitans morsitans* Westwood were bred at the Liverpool School of Tropical Medicine, UK. Tsetse rooms were kept at a temperature of 26–28°C and at 60–80% humidity at all times, with a 12 h/12 h light/dark cycle. The health of all female flies involved in experiments was monitored by recording abortion rates and the weights of offspring (pupae). Females were selected for participation in an experiment when the polypneustic lobes of the larva *in utero* were visible through the female's abdominal cuticle (figure 1*d*), indicating that parturition would occur within the following 24–48 h. For selection purposes, females were kept in a cold room at 2–6°C for no longer than 10 min and were allowed to warm up in the colony room before being transferred to the experimental cage. Each female was used once per experimental condition. We used females of varying ages (between 16 and 60 days old, i.e. between their first and fifth gravidity cycle) to reflect the natural situation as closely as possible. After participating in experiments, females readily fed and continued to produce larvae until they were terminated at 10 weeks old.

### (b) Behavioural experiments

All experiments were done in 60 × 60 × 90 cm pop-up insect cages (Watkins & Doncaster, Leominster, Herefordshire, UK). Cages were initially washed with bleach, and subsequently stained brown using black tea (PG Tips, Unilever UK, London, UK). One wall (60 × 60 cm) of the cage was dark blue, which we used as the floor in the ‘substrate’ and ‘naturalistic’ conditions. In the ‘pentadecane’ condition, the cage was placed horizontally (60 × 90 cm floor area).

Leaf litter was collected at Hampstead Heath (London, UK). To remove compounds contained in the leaf litter that may volatilize at room temperature, and therefore provide an unintended olfactory cue, all leaf litter was sterilized in an autoclave for 2 h at 121°C and subsequently dried at 100°C for 1 h. At these temperatures, soil and surface microbes cannot survive [28], removing the possibility of microbe-generated volatiles. Between experiments, leaf litter was re-sterilized using a drying oven at 121°C for 2 h. The leaf litter consisted mostly of oak leaves (*Quercus* sp., most likely *Quercus robur*) and a small proportion of leaves from other European deciduous trees. Note that the leaves of *Q. robur* have been reported to not emit volatile *n*-pentadecane [29]. Furthermore, a GC–MS analysis of the leaf litter used in our experiments confirmed that no *n*-pentadecane was present.

Natural-colour silica sand (Trustleaf, March, UK) was washed in tap water and then sterilized in an oven at 121°C for 4 h. Between experiments, sand was sterilized again at 121°C for 4 h. Natural-colour sand was chosen to match the leaf litter colours better than white sand to avoid presenting a visually distinct stimulus. Stimuli were presented in 28 × 17.5 × 17 cm food-grade plastic trays (Solent Plastics, Romsey, UK), which each covered approximately 30.7% of the available floor space (electronic supplementary material, figure S1).

Experiments were run over a 2-day period, during which time approximately 80% of females larviposited. On the morning of the second day, stimulus and control trays were exchanged for fresh trays. Stimulus and control trays were placed on the right or left side randomly on day 1, and in reverse order on day 2. Note that we took care to finish these preparations by 13.00 h, so as not to interfere with the flies' period of peak larviposition, which occurred between 14.00 and 18.00 h. Flies were then left unattended for the remainder of the day to keep human presence in the room to a minimum, as savannah tsetse species, particularly females, can be repelled by human odour [30,31].

Pupae deposited in trays were counted every day. To be able to count pupae deposited on the floor, double-sided gel tape (Nano-Grab, Shurtape Technologies, Avon, OH, USA) was used as a separation between the right and left half of the cage floor (electronic supplementary material, figure S1). Pupae were often found stuck to the side of the tape, indicating that the larvae could not cross this barrier. We therefore assumed that pupae found on each side were deposited there, and counted them accordingly.

After 2 days, experiments were terminated and all flies were removed from the cages using a fly aspirator (Katcha Bug Buster, Select IP, Chesterfield, UK).

### (c) Testing the substrate: sand versus leaf litter

To test whether the flies prefer sand or leaf litter as a substrate for larviposition, we first presented 800 ml sterilized sand and 800 ml sterilized leaf litter to gravid female *G. m. morsitans*. Pupae were counted daily. The experiment was repeated 10 times, with 39–51 females added per cage, amounting to a total of 465 females.

### (d) Testing the pheromone: *n*-pentadecane versus paraffin oil

According to Saini *et al*. [5], *n*-pentadecane was the primary active compound of the larviposition pheromone of *G. m. morsitans*. We aimed at replicating this finding in the laboratory, but using leaf litter as a larviposition substrate. To this end, we used a 10^−2^ dilution of *n*-pentadecane (Sigma-Aldrich, Steinheim, Germany; lot no. BCCC7129) in paraffin oil, with a resulting concentration of 7.69 µg µl^−1^. Using 25 µl of this dilution delivered a dose of *n*-pentadecane equivalent to approximately 192 µg—equivalent, approximately, to the amount released by 80 pupating larvae over 2 h [5]. According to Saini and colleagues [5], this dose should be sufficient to show robust attraction of approximately 75% of females, while not being so high as to act as a repellent. As a control stimulus, we used 25 µl of paraffin oil.

Both stimuli were pipetted onto discs of filter paper (Whatman no. 1) and placed in the bottom of a stimulus tray. The tray was then covered with a cardboard-framed mesh that allowed burrowing larvae to fall into the tray below, but prevented flies from entering the tray. Approximately 1 l of leaf litter was then placed on top of the mesh. Stimuli were placed randomly on the left or right side of the cage, and exchanged for fresh stimuli on day 2 as detailed above. Pupae were collected and counted as described above. The experiment was repeated 12 times, amounting to a total of 601 females tested (between 45 and 55 females per cage).

### (e) Naturalistic experiments

To confirm our findings and make them comparable to results obtained from field studies, we conducted an additional experiment aimed at being as close to a natural situation as possible. First, we reasoned that the earliest-depositing female flies on any given day would not encounter freshly pupated larvae in the ground—rather, they would find an area in which larvae deposited the previous day are respiring. Second, the ground would likely be covered in leaf litter or other organic debris. Finally, any additional larva deposited on the same day would add to the complex bouquet of larval and pupal odours that may play a role in attracting females to the site. With this in mind, we designed the following experiment.

Between 14.00 and 18.00 h (peak larviposition time), 20 freshly deposited larvae were collected and placed in a tray containing 800 ml sterilized sand. Most larvae quickly burrowed into the sand, while a small minority pupated on top of the sand. A control tray of larvae-free sand was prepared at the same time. Both trays were covered and left in the fly colony room overnight. The following day (day 2), mesh frames were placed onto both trays, which were subsequently covered in 800 ml sterilized leaf litter. The leaf litter was separated from the sand in this way to prevent flies from touching the sand. Trays were placed into an experimental cage containing 41–52 heavily pregnant females. Also on this day, new stimulus and control trays were prepared by adding larvae to sand as described above.

On day 3, stimulus and control trays were exchanged for freshly made trays and the position of the stimulus and control trays was reversed. All pupae were removed from the trays and from the floor of the cage and counted. On the final day (day 4), trays were removed and all pupae counted. All flies were removed from the cages using a fly aspirator. In total, 573 females were tested in this experimental condition.

### (f) Shade experiment

To gain insight into other factors that may contribute to the females’ larviposition choice, we took advantage of the fact that we had conducted the ‘pentadecane’ experiment placing the cage horizontally, with one side of the cage flanked by a dark blue cage wall. It is well documented that tsetse are attracted to blue surfaces [32,33], and we aimed to test whether this experimental arrangement impacted larviposition choices. Stimulus and control trays were placed on alternating sides of the cage as described above, but for analysis purposes, pupae were grouped according to the half of the cage in which they were recovered—that is, the ‘dark’ side or the ‘light’ side. The analysis was performed for pupae found in the trays and on the floor.

### (g) Chemical analysis

To confirm that the leaf litter did not contain any *n*-pentadecane, we performed gas chromatography coupled with mass spectrometry (GC–MS) on three samples: (i) 0.05 mM pentadecane (Sigma-Aldrich, pentadecane analytical standard, lot no. BCCC7129) in hexane, (ii) 500 µl hexane used to wash a sample of autoclaved and dried leaf litter, and (iii) a hexane blank. Samples were analysed using an Agilent 7890B-5977A GC-MSD in electron ionization (EI) mode. GC–MS parameters were as follows: carrier gas, helium; flow rate, 0.9 ml min^−1^; column, DB-5MS (Agilent); for apolar analyses: inlet, 250°C; temperature gradient, 70°C (1 min), ramp to 230°C (15°C min^−1^, 2 min hold), ramp to 325°C (25°C min^−1^, 3 min hold). Scan range was *m*/*z* 50–565. Data were acquired and analysed using MassHunter software (Agilent, v.B.07.02.1938). Pentadecane peaks were identified based on retention time and extracted ion chromatograms (EIC), as compared with the pentadecane standard.

### (h) Statistical analysis

Pupae deposited in each cage cannot be assumed to be independent, as the presence of a pupa in a stimulus tray may influence the larviposition decision of other females in the cage. Therefore, we calculated a preference index (PI) per cage, given as

$$\mathrm{PI}=\;\frac{n_{\mathrm{stimulus}}-\;n_{\mathrm{control}}}{n_{\mathrm{stimulus}}+n_{\mathrm{control}}}.$$

Thus, PI = 1 indicates that females larviposited exclusively in the stimulus tray, while PI = −1 indicates that all females preferred the control. PI = 0 indicates no preference.

The choice index refers to the preference of females to larviposit on stimulus trays or on the floor. However, as the total available surface area on the floor was larger than on the trays, it was calculated based on the density of pupae found on the floor versus the trays, in pupae cm^−2^.

The PI for the light or shaded half of the cage was calculated in an analogous way to the PI described above, contrasting the number of pupae found on trays and on the floor in the right (shaded) versus left (light) half of the cage.

To compare distributions of pupae across cages within one experimental condition, we used the non-parametric Wilcoxon rank-sum test. Across experiments, we compared distributions using the Kolmogorov–Smirnov test. Analysis of variance (ANOVA) was used to test the effects of other factors that may have influenced the results.

For further statistical analysis, we produced a Bayesian generalized linear mixed-effects model using the Stan language via the R-package *brms* [34]. Samples were drawn from a Bernoulli-type distribution using weakly informative priors (normal distribution with mean 0, s.d. 5). We modelled female choice, binarized to 1 (female chose stimulus/leaf litter) and 0 (female chose control/sand), for the three experimental conditions, using experiment type as population-level effect and cage as group-level effect, resulting in the following model: binary ~ experiment + (1|cage). The model was run across four chains with 1000 warm-up and 4000 post-warm-up draws each. Chains converged according to $\hat{R}$ values ($\hat{R}$ = 1), effective sample size values and visual representations of chain traces. The model is available on Dryad: doi:10.5061/dryad.w0vt4b8w6 [35].

All statistical analysis was done in RStudio using R 4.2.1. Data were plotted using *ggplot2* [36] and *viridis* [37]. Figures were prepared in Adobe Illustrator 24.0.1.

## Data accessibility

All behavioural data and the Bayesian model can be found in the Dryad Digital Repository: https://dx.doi.org/10.5061/dryad.w0vt4b8w6 [35].

Additional data are provided in the electronic supplementary material [38].
